# Gene Flow between the Korean Peninsula and Its Neighboring Countries

**DOI:** 10.1371/journal.pone.0011855

**Published:** 2010-07-29

**Authors:** Jongsun Jung, Hoyoung Kang, Yoon Shin Cho, Ji Hee Oh, Min Hyung Ryu, Hye Won Chung, Jeong-Sun Seo, Jong-Eun Lee, Bermseok Oh, Jong Bhak, Hyung-Lae Kim

**Affiliations:** 1 Syntekabio Inc., Seoul, Korea; 2 DNA link Inc., Seoul, Korea; 3 Korean Bioinformation Center, KRIBB, Daejeon, Korea; 4 University of Science and Technology, Daejeon, Korea; 5 Division of Structural and Functional Genomics, Center for Genome Science, Korea National Institute of Health, Seoul, Korea; 6 Department of Obstetrics and Gynecology, College of Medicine, Ewha Womans University, Seoul, Korea; 7 Department of Biochemistry and Molecular Biology, Seoul National University of Medicine, Seoul, Korea; 8 Department of Biomedical Engineering, Kyung Hee University College of Medicine, Seoul, Korea; University of Wisconsin, United States of America

## Abstract

SNP markers provide the primary data for population structure analysis. In this study, we employed whole-genome autosomal SNPs as a marker set (54,836 SNP markers) and tested their possible effects on genetic ancestry using 320 subjects covering 24 regional groups including Northern ( = 16) and Southern ( = 3) Asians, Amerindians ( = 1), and four HapMap populations (YRI, CEU, JPT, and CHB). Additionally, we evaluated the effectiveness and robustness of 50K autosomal SNPs with various clustering methods, along with their dependencies on recombination hotspots (RH), linkage disequilibrium (LD), missing calls and regional specific markers. The RH- and LD-free multi-dimensional scaling (MDS) method showed a broad picture of human migration from Africa to North-East Asia on our genome map, supporting results from previous haploid DNA studies. Of the Asian groups, the East Asian group showed greater differentiation than the Northern and Southern Asian groups with respect to Fst statistics. By extension, the analysis of monomorphic markers implied that nine out of ten historical regions in South Korea, and Tokyo in Japan, showed signs of genetic drift caused by the later settlement of East Asia (South Korea, Japan and China), while Gyeongju in South East Korea showed signs of the earliest settlement in East Asia. In the genome map, the gene flow to the Korean Peninsula from its neighboring countries indicated that some genetic signals from Northern populations such as the Siberians and Mongolians still remain in the South East and West regions, while few signals remain from the early Southern lineages.

## Introduction

The X and Y chromosomes and mitochondrial DNA (mtDNA) recombine to a much lesser extent than do autosomes [1]. This limited, or non-existent, recombination in haploid SNPs (single nucleotide polymorphisms) has been used to trace the evolutionary lineages of individuals and populations showing either maternal or paternal transmission. However, obstacles encountered with the use of lesser (or non-) recombining SNPs, such as genetic drift, low polymorphic nature, natural selection, and the small size of these non-recombining DNAs, limit the scope of genetic studies. Recently, an advanced technology, termed whole genome sampling analysis, has enabled the genotyping of nearly one megabyte of diploid autosomal SNPs at a time [2]. These autosomal SNPs are a reservoir for human evolutionary history, nearly 100 times larger than that of lesser (non-) recombining SNPs, and include the entire range from highly conserved to highly polymorphic SNPs. However, when individuals and populations of different races and regions are compared using the whole genome approach, a large proportion of the redundant and recombining regions exacerbate the difficulties encountered in exploring evolutionary relationships, common traits and disease susceptibilities between populations.

The origin of Amerindians, based on Y chromosome and mtDNA studies, revealed that they migrated from Siberia to Alaska [3]. Recently, over 600 autosomal short tandem repeat (STR) markers were used to delineate the migration routes of most of the Amerindians [4], while analysis of molecular variance, inferred from metric distances between DNA haplotypes of human mitochondrial DNA, was used for migration studies of Southern and Northern Europe [5]. Some diabetes-related rare markers were also found in Amerindian and Finnish families [6]. Y chromosomal DNA haplogroups disclosed the dual origins of the Korean population [7], while the origins of East Asians have been studied using certain autosomal loci [8]. Investigation into male demography in East Asia using Y chromosome haplogroups in a simulation analysis provided information on the settlement times of Northern and Southern populations [9]. Recently, genome-wide analysis of haplotypes in Asia was reported that more than 90% of East Asian haplotypes could be found in either Southeast Asian or Central-South Asian populations and showed clinal structure with haplotype diversity decreasing from south to north [10].

Given a symmetric distance (or a covariance) matrix between individuals, the dimension-reduction methodologies, such as multidimensional scaling (MDS) and principal components analysis (PCA), can display individual interacting patterns effectively by means of two, or more, major eigenvector components [11]. One of the relationship matrices for the comparison of individual whole genomes is based on identity-by-state (IBS) distances that show an opposite relationship to allele-sharing distances (ASD) [12] when applied to a neighbor-joining (NJ)-based phylogenetic tree [13]. Compared to the dimension-reduction methodologies described above, the Markov Chain Monte Carlo (MCMC)-based STRUCTURE program is also one of the most popular methods for understanding genetic structures and admixtures [14]. Recently, Salmela et al. [15] showed that 250K genome-wide autosomal SNPs could provide powerful resources for the study of population structure and differentiation in Northern Europe, including Northern Germany, Great Britain, Finland and Sweden.

**Figure 1:**
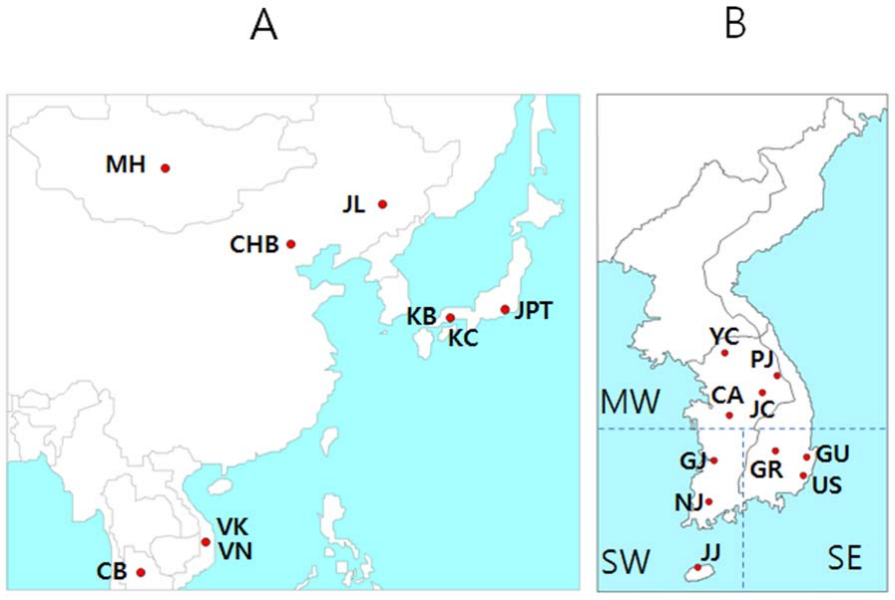
Map of Asia (A) and the Korean Peninsula (B). Historically and geographically, the Korean Peninsula can be subdivided into two regions, the West and East, based on the continuous ridge-line of mountains from Janggun Peak of Mt. Baekdu on the Northern border of the Korean Peninsula down to Mt. Jiri near its Southern end. The Western part of South Korea is further subdivided into Middle and South regions. The abbreviations for populations, their geographical regions and sample sizes are summarized in Table 1.

Geographically, the Korean Peninsula is a strategic location in East Asia surrounded by China, Japan and Russia. Therefore, the objectives of our gene flow analyses in Northern, Eastern and Southern Asia were to determine the extent of the gene flow from these neighboring countries and the levels of genetic signals that have remained in the Korean Peninsula, using subjects collected from the ten most historical regions in South Korea. In addition, samples from Mongolia, Jinlin in China and Amerindian populations, representing Northern people; Cambodians and Vietnamese, representing Southern people; and Chinese and Japanese were used to measure their influences on the gene flow. For this study, we propose three different gene flow models based on historical events, geographical location and anthropology; Model I: SW (South West) Korea, which includes the JJ, NJ and GJ regions (Fig. 1) has some lineages in common with the BaekJae Empire (BC18-AD660) through a migration event by the Northern people of the Goguryeo Empire which ruled most of Northern Manchuria and Korean peninsula (BC37-AD568), and are also closely related to Mongolians. They ruled most of the Eastern part of China and most of the South Western part of the Korean Peninsula. Model II: SE (South East) Korea, which includes the GU, US and GR populations (Fig. 1), is the region where the Northern people from Siberia (based on their grave patterns and ancient cultural traditions) and the loyal Southern families were settled in the Shilla(BC57-AD918) and Kaya(AD42-532) Empires, respectively. Model III: MW (Middle West) Korea, which includes the YC, JC, CA and PC populations (Fig. 1), is the human melting pot of the Korean Peninsula where Western Chinese, including those from the SanDung Peninsula, crossed the Yellow Sea between China and Korea, living and trading in both regions. In addition, it is known that historically the Northern and Southern Koreans often clashed with each other in this region. The geographical locations of the models used in this study are shown in Fig. 1 and Table 1.

**Table 1:** Geographic origins and sizes of the samples.

Sample nationality (or Ethnicity)	Sample name regions (abbreviations)	Sample size ( = 320)
South East (SE)Korean	Gyeongju(GU)	16
	Goryeong(GR)	15
	Ulsan (US)	16
Middle West (MW)Korean	Jecheon (JC)	16
	Yeoncheon (YC)	16
	Cheonan (CA)	16
	Pyeongchang (PC)	16
South West (SW)Korean	Gimje (GJ)	15
	Naju (NJ)	16
	Jeju (JJ)	16
South Korean	Vietnamese-Korean (VK)	8
Japanese	Kobe(KB)	4
Korean-Japanese	Kobe(KJ)	6
Japanese	^1^HapMap(JPT)	16
Chinese	Jilin (JL)	16
Chinese	^1^HapMap (CHB);	16
Vietnam	Kinh (VN)	16
Cambodia	Khmer (CB)	16
Mongolia	Khalkha (MH)	16
American Indian	^2^Amerind (AI)	16
America	^1^HapMap (CEU)	16
Nigeria	^1^HapMap (YRI)	16

*Samples with N<16 were not used for the statistical analysis purpose. The genotype data (^1^HapMap and ^2^Amerind: 80 samples) were collected from HapMap and Affymetrix Inc., respectively, and the rest (240 samples) were genotyped in this study.

Marker selection is the most important step in data handling. Because sex chromosomes and mtDNA recombine to a much lesser extent than do autosomes during meiosis, they do not record gene flow event on themselves. Only when complete enumeration or meaningful sampling survey was used, considerable inferences can be deducted. On the other hand, because autosomes accumulate the record of the gene flow event by way of the recombination, the markers on autosomes can be a better object than the ones on sex chromosomes and mtDNA; however, it is also necessary to deal with the effects of recombination hotspots (RH) and linkage disequilibrium (LD). Therefore, in this study, we have evaluated several variables including RH, LD and genotype call rates using known clustering methods such as IBS-based Multi-Dimensional Scaling (MDS), Principal Components Analysis (PCA), the ASD-based Neighbor Joining (NJ) method, and STRUCTURE using 320 subjects from Amerindian populations, from four populations from the HapMap Project (Yoruba, Caucasian, Japanese, and Chinese), and from Vietnamese, Cambodians, North-Eastern Chinese, Mongolians, and Koreans to reconstruct a human migration route that had been partly analyzed using mtDNA and the X and Y chromosomes.

## Results

### Genome map of human migration

**Figure 2:**
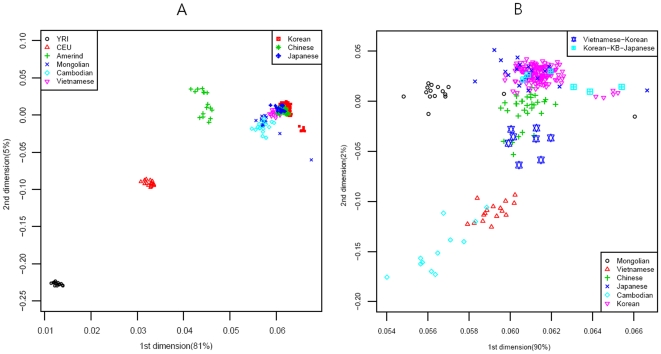
Genome Map of World (A) and Asia (B).

The genome map was drawn using MDS with an RH-free dataset (Supporting Methods and Discussion S1). The genome map in Fig. 2A showed that the Yoruban, Caucasian, and Amerindian populations lined up first, and then Northern and Southern Asian populations were merged in East Asia in Fig. 2B. In the extended genome map for Northern and Southern Asia, the merged pattern became clearer than that of the whole populations where the MH (Mongolian) population lined up to JPT (Tokyo) and JJ (JeJu) populations, while the CB (Cambodian) population lined up to the VN (Vietnamese), VK (Vietnamese-Korean), CHB (Peking), and JL (Jilin) populations.

**Figure 3:**
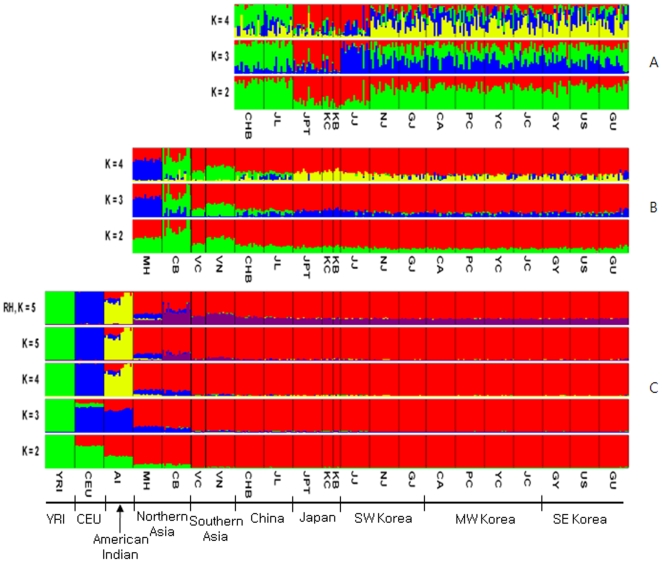
Genetic Structure. A)World, B)Asia, C)Korea, Japan and China. Note that the plot with a recombination hotspot (RH) of K = 5 on the left is shown to compare with the same plot without the RH effect on the right where the purple bands were spread over all Asian populations.

In our analysis of genetics structure by STRUCTURE program [16], four populations displayed perfect population membership probabilities, the Africans, Caucasians, Amerindians, and Northern Asians, and three populations displayed partial membership, the Mongolians, Vietnamese, and Cambodians (Fig. 3). Substantial admixture patterns between populations were also observed for Mongolians and Caucasians, and Amerindians with East Asians and Caucasians.

### Gene flow between the Korean Peninsula and neighboring countries

**Figure 4:**
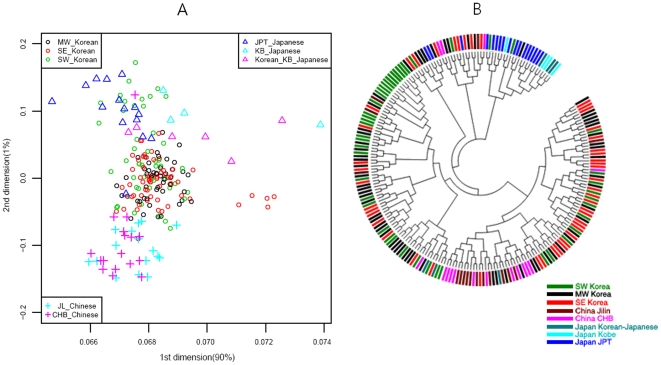
MDS and NJ Tree of Korean, Chinese and Japanese. A)MDS(multi-dimensional scaling) [17], B)NJ Tree. In the NJ tree, samples for Japan, Kobe-Japan, SW Korea, SE Korea, MW Korea, China, Jinlin, and Korean-Japanese are blue, cyan, green, purple, black, brown and dark green, respectively.

Genome map and an NJ tree [18], [19] for Korea-China-Japan are shown in Fig. 4, where the genome map is a further extension of the World and Asian map (Fig. 2). In the figure, dots representing the Chinese are located at the bottom and those for Japanese are at the top, while some outliers in SE Korea and Kobe in Japan are shown on the right hand side. Of the ten historical regions in South Korea, some in SW Korea overlap with those of Japan, while most of the MW Korean regions are located at the center of the genome map. Similarly, in the NJ tree, nodes for SW Korea are close to those in Japan, MW Korea is close to China, and SE Korea is located at the right hand side of the tree.

The gene flow events of the three selected models for SW, MW and SE Korea can be assessed using the genome map. The populations in Model I (SW Korea) are closer to Mongolians than are the other two regions in the genome map (Fig. 2B). Historically, some of the loyal families and their subjects in the Goguryeo Empire moved to this region and formed the BaekJae Empire in BC18-22. This region also showed connections with populations in Tokyo (JPT), as illustrated in Fig. 4. Certain outliers in Model II (SE Korea) display some similarity to the people of Kobe, a port city near Osaka, indicating that there may have been links between the two regions. In addition, considering that the SE Korea region has some connections with Siberian lineages, with respect to grave patterns and culture, it is possible that the outliers in the GU and Kobe (KB) populations could be of Siberian lineage. On the other hand, the GR and US populations showed average signals in the Korean Peninsula. Historically, the Kaya Empire, with its southern lineages, was formed in the GR region and then the Shilla and Kaya Empires became united around AD532. Very recently, the US region became one of the rapidly developing regions, and people from other provinces moved to this region. This might explain why it shows an average signal in South Korea. Model III (MW Korea): the Middle West area formed a melting pot in the Korean Peninsula because populations moving from South to North, North to South, and from Eastern China, including the SanDung peninsula, to the Middle West in Korea all came together in this region. In the genome map, the signals for MW Korea are also close to those for Peking (CHB) in China. The overall result for the Korea-Japan-China genome map indicates that some signals for Mongolia and Siberia remain in SW Korea and SE Korea, respectively, while MW Korea displays an average signal for South Korea.

### Genetic drift in the Korean Peninsula

Fst statistics assume that alleles arrive in the population via migration rather than mutation. Therefore, the mean Fst of two populations displays the differentiation caused by a migration event. Table 2 indicates that the population showing the highest level of differentiation in the world (in our dataset) was the Amerindian group, while, in Asia, the East Asian group revealed the highest level of differentiation compared to Northern and Southern Asians, whereas there was no difference among East Asians, including Koreans, Japanese and Chinese. In addition, Salmela et al. [15] suggested that Eastern Finns also showed signs of genetic drift by analyzing the relationships between monomorphic markers. Using the same measurements for East Asian groups (Korea, Japan and China), nine out of ten historical regions in South Korea showed similar signs of genetic drift that might be due to the later settlement of East Asia. Of the nine historical regions, Gimje (GJ) showed the highest genetic drift. On the other hand, Gyeongju (GU) in South East Korea and Jinlin (JL) in North-Eastern China displayed the lowest drift, suggesting that the populations in the GU and JL regions could be earlier settlers of the Korean Peninsula (Table S1 in supporting information).

**Table 2:** Mean Fst distribution of World, Asia and East Asia.

World mean Fst		Asia mean Fst		East Asia mean Fst	
AI	0.2954	East Asia	0.0850	KOR	0.0100
YRI	0.1995	South Asia	0.0635	CHB	0.0099
East Asia	0.1601	North Asia	0.0118	JPT	0.0100
CEU	0.1180	-	-	-	-
South Asia	0.0188	-	-	-	-

East Asia: Korea, Japan, China. South Asia: Cambodia, Vietnam. Northern Asia: Mongolia. Three mean Fst(s) by STRUCTURE was computed in World, Asia, and East Asia, respectively.

## Discussion

Current populations in the Korean Peninsula were formed through interactions between populations from Southern and Northern Asia. Therefore, the goal of this study was to determine whether there are still genetic signals remaining in the Korean Peninsula using autosomal genome-wide SNPs, which are 100 times more frequent than those in lesser recombining DNA, such as mtDNA, X and Y chromosomes. Interestingly, some of the historical regions and the regionally separated island, Jeju (JJ) and Gyeongju (GU) in Korea still harbor some old signals from Northern populations, while few signals remain from Southern populations.

In this study, we reconstructed the genome map using data from 320 individuals in 24 groups after correcting and re-evaluating all bias parameters (Supporting Methods and Discussion S1, Fig. S1, S2, S3 and S4). Interestingly, the genome map was similar to the physical map of Europe and Asia, a relationship that was reported previously in a correlation experiment between physical distances and genetic variations by Mountain and Cavalli-Sforza [12]. In addition, genetic structure analysis using the STRUCTURE method revealed the existence of five major populations, African, Caucasian, Amerindian, North-East Asian, and Southern Asian. Therefore, in between, there were significant admixtures such as Mongolian with Caucasian, Vietnamese (or Cambodian) with unknown Southern original settlers, and Amerindians with both North-East Asians and Caucasians (Fig. 2). Thus, the genome map could be used to estimate the relative evolution times between populations and genome interactions (or gene flows) (Fig. 4) such as Korean-Vietnamese, between Vietnam and Korea; Korean-Japanese, between Korea and Japan; Mongolian between Amerindians and North-East Asians; Chinese between Koreans and Vietnamese; and Vietnamese between Cambodians and Chinese. In particular, through both the genome map and STRUCTURE results, the evolutionary relationship between Koreans, Japanese, and Chinese has become clearer since it can be seen that the North-East Chinese (JL) and the Peking Chinese (CHB) are relatively closely related, and the Japanese (JPT) and Korea-JeJu (JJ) are also fairly close (Fig. 2).

In this study, we didn't include North Korean samples because of political reasons. However, historical migration event of BaekJae from Goguryeo Empire (BC37-AD568) in Northern Korea imply that Northern lineages remain in South Korea. Nevertheless, considering the fact that recent migration events between provinces in North Korea are much less than those in South Korea, the gene flow study of North Korean may provide additional clues in the future study.

## Materials and Methods

### Ethics Statement

DNA samples of Southern and Northern Asian groups for SNP genotyping were obtained from the Biobank of the National Genome Research Institute (BNGRI), and from the Northern East Asian Nation Functional Genome Project. The samples used for this study all provided written informed consent.

### Recombination hotspots

HapMap Phase II [21] provides highly dense SNPs compared to most of the 50K Affymatrix chip SNPs used in this study. Therefore, given a marker position in the 50K Affymatrix genotype data, all lower and upper 2.5K SNPs around each of the 50K SNP markers were extracted from HapMap and named the 5K_HapMap dataset. Using the 5K_HapMap dataset, the pairwise R^2^ coefficients and their means in each of the four populations, CHB, JPT, CEU, and YRI, were calculated at each marker position using the EM algorithm [22]. Therefore, each of the 50K SNP markers has four different mean R^2^ coefficients, and if at least one of the four populations (CHB, JPT, CEU, and YRI) had an R^2^ value less than 0.7, the marker position was considered as a possible recombination hotspot (RH). Overall, 5K of 54K SNPs were assigned as recombination hotspots. The extraction of 5K spanning SNPs from HapMap and exhaustive pairwise LD (linkage disequilibrium) calculations for the selected 5K_HapMap dataset were performed on an IBM supercomputer (Model JS20) and a cluster computer (DualPowerPC970) with 100 nodes, and took four working days with most of the nodes.

### Generation of different GCR sample sets

The average genotype call rate (GCR) of the current data is 98.7% and named as “data0”. To generate several sample sets having a series of different successful call rates, no call genotypes were inserted randomly in each sample:
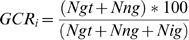
(1)where i is a sample index, Ngt, Nng, and Nig are the numbers of genotypes, natural no call genotypes, and randomly inserted no call genotypes, respectively, and the sum of Ngt, Nng, and Nig is the total number of SNPs used in this study. In this way, three extra genotype datasets, data1, data2, and data3, were generated as different call rate datasets, GCR (95%), GCR (90%), and GCR (85%), respectively. The marker call rate cutoff of data0 was set to 95% and those of data1, data2 and data3 were set to 80%.

### Regional specific markers

The selection of regional specific markers (RSM) was performed using variance analysis. The variance (var_i_) for RSM can be computed as:
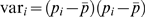
(2)

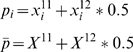
(3)

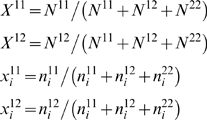
(4)where i is a population, n11, n12, and n22 are the number of genotypes in a population, N11, N12 and N22 are the number of genotypes in the whole populations, and p is the allele frequency. The allele frequency in a population and the mean allele frequency for all populations are shown in equation (3). At each marker position, the variances of allele frequency in each population were computed using equations (2) to (4), and then one or more variances that are relatively different from the rest of the populations could be calculated using the z-scores of the variances:
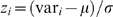
(5)where μ and σ are the mean and standard deviation of the variances over all populations at each marker position, respectively. Among the z-scores at a marker position, the marker with at least one population (z-scores >4.0 or <−4.0) was selected. Here, the z-score with 4.0> or <−4.0 indicates that the marker variance of a specific population is significantly greater, or smaller, than the variance mean. Of the selected markers, the first one of two, or more, consecutive markers in 500K, or less distance, was selected and used. In a visual inspection, most of the markers selected with LD (linkage disequilibrium) blocks were located within 200K SNP intervals or less. Therefore, these RSMs are considered as LD-free marker datasets.

### Subjects and Genotyping

A total of 320 subjects from 24 regional groups were analyzed in this study. They include Yoruba (YRI), European (CEU), Japanese (JPT), Chinese (CHB), Amerindians (AI), and several population groups from Southern and Northern Asia comprising Chinese from the Jilin area (JL), Vietnamese (VN), Cambodians (CB), Mongolians (MH), and Koreans from ten cities in South Korea (Table 1).

Autosomal SNP genotyping data for the YRI, CEU, JPT, and CHB regional groups were obtained from the International HapMap database (www.hapmap.org) and Amerindian data (AI) were obtained from Affymetrix. Of these, the genotype call rates were sorted, and the best 16 samples from each population of 45 unrelated samples for the JPT and CHB groups (or 60 for the YRI and CEU groups) were selected for this study. For the Southern and Northern Asian groups, genotyping was performed with the Affymetrix GeneChip Mapping 50K_Xba array that comprises 58,960 SNPs, according to the manufacturer's protocol (Affymetrix, Santa Clara, CA, USA). The BRLMM algorithm (Bayesian Robust Linear Model with Mahalanobis distance classifier) [23], [24] was applied to genotype calling for one batch of all samples after initial genotyping with the Dynamic Model algorithm [25]. For the selection of samples in our study, several sample folds with their ages and residential histories were collected to determine whether they were native residents.

## Supporting Information

Supporting Methods and Discussion S1 (0.07 MB DOC)

Table S1 Fst, IBS, and Monomorphic marker.
(0.07 MB XLS)

Figure S1 MDS without recombination hotspot (RH) filtering. MDS plots with SNPs after QC, QC+500KB spacing between SNPs, and using only regional specific markers (RSM) are shown in columns. Different genotype call rates (98% to 85%) are shown in rows.
(0.06 MB TIF)

Figure S2 MDS with recombination hotspot (RH) filtering.
(0.06 MB TIF)

Figure S3 PCA without recombination hotspot (RH) filtering. MDS plots with SNPs after QC, QC+500KB spacing between SNPs, and only regional specific markers (RSM) are shown in columns. Different genotype call rates (98% to 85%) are shown in rows.
(0.06 MB TIF)

Figure S4 MDS with recombination hotspot (RH) filtering.
(0.06 MB TIF)
