# Supplementary material for: Gene Flow between the Korean Peninsula and Its Neighboring Countries
Source: PLoS One. 2010 Jul 29;5(7):e11855. doi: 10.1371/journal.pone.0011855 (PMC2912326; doi:10.1371/journal.pone.0011855)
Supplement: Supporting Methods and Discussion S1 — (0.07 MB DOC) [file pone.0011855.s001.doc]

Supporting Methods & Discussion S1

**Clustering algorithms and methods**

IBS (identical by state) similarity coefficients and ASD [12] (Mountain and Cavalli-Sforza 1996) between two individuals (i,j) are summarized as:

$ASD_{ij}=\frac{n2+n1*0.5}{n0+n1+n2}$ (1)

$ASD_{ij}^{'}=\frac{n2+n1*0.5}{\max}$ (2)

$IBS_{ij}=1-ASD_{ij}$ (3)

$IBS_{ij}^{'}=1-ASD_{ij}^{'}$ (4)

where n0, n1, and n2 are the number of genotype pairs, AA:AA, AA:AB, and AA:BB, respectively, between two individual diploid genomes and max is the maximum value among numerators in equation (1). The range of numerators in equation (2) is about 0.0 ~ 0.3 in our data set. Therefore, equation (4) has the data range of 0.0 ~ 1.0 while that of equation (3) ranges from 0.7 ~ 1.0.

An approximated covariance between two individuals (i,i’) [11] is summarized as:

$Cov_{ii'}=\frac{1}{N}MM'$ (5)

$M_{ij}=\frac{C(i,j)-\mu(j)}{\sqrt{p(j)(1-p(j))}}$ (6)

where *C(i,j)* is the number of variant alleles for a marker *j* and an individual *i* (or *i’*), *μ(j*) is the mean of *C(i,j)* over all individuals at a marker position, and *p(j)* is an estimate of allele frequency (=*μ(j)*/2).

The posterior probability of population membership of an individual (i) using the MCMC algorithm [14] is summarized as:

$\Pr(z_{i}=k|X,P)=\frac{\Pr(x_{i}|P,z_{i}=k)}{\sum_{k'=1}^{K} \Pr(x_{i}|P,z_{i}=k)}$ (7)

$\Pr(x_{i}|P,z_{i}=k)=\coprod_{l=1}^{L} p_{kl(i)}q_{kl(i)}$ (8)

where p_kl_ (or q_kl_) is the frequency of allele x at locus l in an individual i and population k, and z_i_ is a population from which the individual i originates.

Using IBS scores and covariance matrices in equations (4) and (6), respectively, a dimension-reduction procedure known as the maximizing Eigen value was performed by the Jacobi transformation routine given by Press and colleagues [17]. In addition, the STRUCTURE method with an admixture model was performed with the parameter set: K=2 to 5, burn-in=10,000, and replication=10,000. Neighbor joining trees were generated with MEGA 4.0 [19] using ASD among individuals [12, 19]. To express phylogenetic trees in a more intuitive way, color-branched trees were generated with the tool HyperTree [20].

The Euclidian distances between standard (call rate >=98%) and turbulent (call rate <=95%) coordinates from the plots created by the MDS and PCA methods were calculated and their root mean square deviations (RMSD), known as standard errors, can be calculated as shown below:

$d_{k}=\sqrt{(x_{k}^{A}-x_{k}^{B})^{2}+(y_{k}^{A}-y_{k}^{B})^{2}}$ (9)

$RMSD=\sqrt{\sum_{k=1}^{n} (d_{k}-\mu)^{2}}/n$ (10)

where n is the number of subjects, k is the serial number, and μ is the mean of the distances. Pairwise *Fst*, *Fit* and *Fis* of populations were calculated. *Fst* is the genetic divergence among subpopulations, *Fit* is the total departure from the Hardy-Weinberg Equilibrium, including that within and among subpopulations and *Fis* is the departure from Hardy-Weinberg Equilibrium in local subpopulations. In addition, the pairwise mean IBS (identical by state) values and monomorphic markers in each population were calculated and are shown in Supporting Table 1. The basic genetic parameters such as *Fst* statistics, IBS values in equation (3), monomorphic markers and MDS were computed using a genetic software package, IDA (index data analysis), and PCA was performed using the PCA tool [11].

**Statistical analysis of the effect of recombination hotspots in the genome map**

We estimated the influence of recombination hotspots for autosomal DNA. First, we generated the genome maps using the two eigenvector components (x and y coordinates) of the MDS and PCA methods with all SNPs and a subset of SNPs (500K spacing for the control of LD effect and regional specific markers, RSMs). Second, the Euclidian distances between standard (98% call rate) and turbulent datasets (95%, 95% and 85%) were calculated, and their RH effects were measured in terms of standard errors using equations (9) and (10). The results are summarized in Table S2. Interestingly, compared to the reference data, the standard errors within the datasets, after removing the RH candidate markers, were significantly reduced in both PCA and MDS, implying that recombination hotspots trigger errors in genome maps drawn by dimension reduction methodologies. In addition, the PCA plot is much more tolerant than the MDS plot in terms of error rates, which implies that PCA and MDS use two different data processing methodologies with respect to genome-wide SNP data.

**Variable dependency in clustering methods**

We performed genetic structure analysis of 54K SNPs from 320 subjects in 24 regional groups using the STRUCTURE program and evaluated the dependencies of three different variables, the recombination hotspots (RH), genotype call rates (GCR), and regional specific markers (RSM) (see Materials and Methods). When the whole 54K SNP dataset was used, there were two color bands for the Koreans, Japanese, and Chinese, but after removing 5K of the RH candidates from the 54K SNPs, the second band for those populations disappeared (Fig. 3), implying that the noise from the recombination hotspot SNPs produced the extra color band. In addition, lowering the genotype call rate from GCR (98%) to GCR (85%) meant that the STRUCTURE result was more tolerant (data not shown).

Using the two eigenvector components of MDS and PCA, genome maps were plotted to evaluate the dependencies of the three variables RH, GCR and RSM (Fig. S1-S4). There were small differences between the genome maps with RH and non-RH, using the MDS, PCA, and NJ methods, but the variable dependency patterns between PCA and MDS showed greater differences. For example, in the MDS method, the overall structure and stability of the genome map was affected by GCR, RH, and RSM variables, whereas for PCA, the genome map was only affected by the RSM variable. The result indicates that the MDS method is not tolerant of missing (or false) genotype data, while PCA will tolerate the same variables. In addition, the overall patterns of the maps with RSMs also differ between MDS and PCA, where MDS and PCA have four and three clusters, respectively (Fig. S3-S4). Overall, the visual analysis of the variable dependencies of the various methods can be summarized as follows: PCA is not stable with respect to Linkage Disequilibrium effects, while the STRUCTURE, MDS, and NJ methods are relatively tolerant of LD effects. On the other hand, MDS, NJ and STRUCTURE are not stable with respect to RH and GCR effects, although they are stable for the LD effect, whereas PCA is relatively tolerant of RH and GCR effects.

Table S2. Root mean square deviation (RMSD) of distances between two genome maps*

| Data set  (call rate %) | MDS  RH No RH | | | | PCA  RH No RH | | | |
| --- | --- | --- | --- | --- | --- | --- | --- | --- |
|  | ^+^SE | mean | ^+^SE | mean | ^+^SE | mean | ^+^SE | mean |
| All(98vs95) | 0.541 | 0.352 | 0.032 | 0.04 | 0.034 | 0.041 | 0.036 | 0.04 |
| All(98vs90) | 0.696 | 0.813 | 0.08 | 0.08 | 0.049 | 0.06 | 0.042 | 0.053 |
| All(98vs85) | 1.772 | 2.066 | 0.528 | 0.355 | 0.061 | 0.078 | 0.048 | 0.058 |
| 500k(98vs95) | 0.54 | 0.351 | 0.049 | 0.068 | 0.04 | 0.056 | 0.039 | 0.047 |
| 500k(98vs90) | 0.681 | 0.802 | 0.096 | 0.101 | 0.054 | 0.074 | 0.046 | 0.059 |
| 500k(98vs85) | 1.724 | 2.045 | 0.526 | 0.356 | 0.062 | 0.082 | 0.051 | 0.064 |
| RSM(98vs95) | 0.512 | 0.515 | 0 | 0 | 0 | 0 | 0 | 0 |
| RSM(98vs90) | 0.519 | 0.789 | 0.311 | 0.208 | 0.356 | 0.238 | 0.129 | 0.192 |
| RSM(98vs85) | 1.913 | 2.411 | 0.661 | 0.391 | 0.378 | 0.25 | 0.164 | 0.231 |

+SE: standard error. The SEs in brown are reduced to those in red for both MDS and PCA. All: all data sets after QC. 500K: all consecutive two-marker distances less than 500K or lower were removed from All data sets. RSM: of the 24 regional groups, if there was at least one regional marker with Z scores >= 4.0, the marker was kept and the rest of the markers were removed. *RMSD: The root mean square deviation (RMSD) (also root mean square error (RMSE)) is a frequently-used measure of the differences between values predicted by a model, or an estimator, and the values actually observed from the entity being modeled or estimated. *The reference and testing genome maps are shown in supporting information Figs. S1-S4.

**Discussion**

Using the PCA method for population analysis, the covariance matrix is formulated as E[(X-EX)(Y-EY)] where EX (or EY) is the mean of genotypes over all markers for a population X (or Y). On the other hand, using PCA for individuals given in equations (3) to (4) (see Materials and Methods), it should be dealt with as a simplified approximation where the covariance matrix is E[(X-EM)(Y-EM)/(genetic drift parameter)] and the term EM is the mean of genotypes over all populations at each marker position. Therefore, by the formula itself, PCA for individuals is designed to be highly sensitive to the independence of markers (for example, the linkage disequilibrium effect) because it is a marker-based covariance matrix (Fig. 2). However, for the MDS method, the dimension-reduction part is the same as for PCA, but the similarity (or distance) matrix can be used instead. For example, when an IBS score is used for genome-wide autosomal SNPs in the MDS procedure, the IBS similarity matrix for individuals can be used directly for stratifying populations since the individual relationships, using the IBS score, can be delineated proportionally. When comparing similarity between individual genomes, the MDS method could be more sensitive to missing (or false) genotypes than the covariance of PCA, because in their similarity matrices, each matrix element contains all the missing (or false) genotypes, without a statistical filtering process such as the covariance in PCA. However, MDS is less influenced by the independence of marker distances than PCA, since the IBS score is based on the similarity of the whole genome. In addition, the ASD (allele sharing distance)-based NJ (neighbor joining) method is also related to a property of the IBS score, since ASD is 1 – IBS in equations (1) to (2) (see Materials & Methods). The ASD-based NJ tree also displayed the difference in phylogenetic trees and relationships between individuals. The merit of STRUCTURE is that it can provide individual ancestry information and posterior probability of population memberships. It has been debated whether STRUCTURE could be applied for genome-wide autosomal SNPs as the authors caution against assumptions based on the independence of markers (for example, Linkage Disequilibrium effects) [14]. However, the debate is not so critical for MCMC-based STRUCTURE because MCMC is related to the comparison of distribution between individual genomes using the prior information of assumed K-clusters in equations (5) to (6).

Overall, when genome-wide autosomal SNPs are used, the four methods described above can be classified into two groups: the genome similarity-based methods (MDS, ASD-NJ, and STRUCTURE) and the independent marker similarity-based method (PCA). The only factors that could influence the accuracy of population clustering for the first group are the number of markers creating information content and the number of missing (or false genotype) markers producing information dilution, while for the second group, the effects of marker independence (for example, Linkage Disequilibrium effects) may be influential. In addition, when genome-wide autosomal SNPs are used for genetic studies, dependent variables such as RHs, RSMs and GCR should be adjusted accordingly because they could influence prediction, simulation, and population clustering by random noise SNPs, LD effects, and missing (or false) genotypes. For recombination hotspot SNPs, an underlying hypothesis is that the recombination hotspot has been identified from a founder, even though an additional or a new hotspot could be generated as time passes. Therefore, if a marker position is a recombination hotspot for all four populations in the HapMap project (YRI, CEU, CHB, and JPT), then it is highly likely that the position was a recombination hotspot in the founders. The STRUCTURE program distinguished the recombination-hotspot-free SNPs from the whole genotype data (Fig. 2). The same RH SNPs were also partially distinguished by the other clustering methods such as PCA, MDS, and NJ. Overall, the visual and statistical analysis of the variable dependencies indicates that RH, GCR, and RSM should be considered as dependent variables when genome-wide autosomal SNPs are used for genetic studies.

**Supporting Figures & Table Legends**

Figure S1. MDS without recombination hotspot (RH) filtering.

MDS plots with SNPs after QC, QC+500KB spacing between SNPs, and using only regional specific markers (RSM) are shown in columns. Different genotype call rates (98% to 85%) are shown in rows.

Figure S2. MDS with recombination hotspot (RH) filtering

Figure S3. PCA without recombination hotspot (RH) filtering

MDS plots with SNPs after QC, QC+500KB spacing between SNPs, and only regional specific markers (RSM) are shown in columns. Different genotype call rates (98% to 85%) are shown in rows.

Figure S4. MDS with recombination hotspot (RH) filtering.

Table S1. *Fst*, IBS, and Monomorphic markers.

Table S2. Root mean square deviation (RMSD) of distances between two genome maps*
