# Supplementary figures and images for: Gene Flow between the Korean Peninsula and Its Neighboring Countries
Source: PLoS One. 2010 Jul 29;5(7):e11855. doi: 10.1371/journal.pone.0011855 (PMC2912326; doi:10.1371/journal.pone.0011855)

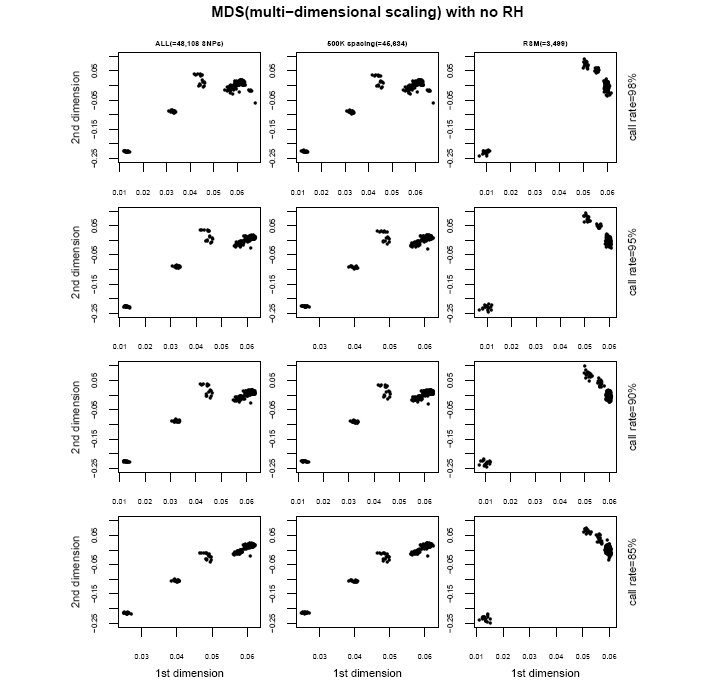

Supplement: Figure S1 — MDS without recombination hotspot (RH) filtering. MDS plots with SNPs after QC, QC+500KB spacing between SNPs, and using only regional specific markers (RSM) are shown in columns. Different genotype call rates (98% to 85%) are shown in rows. (0.06 MB TIF) [file pone.0011855.s003.tif]

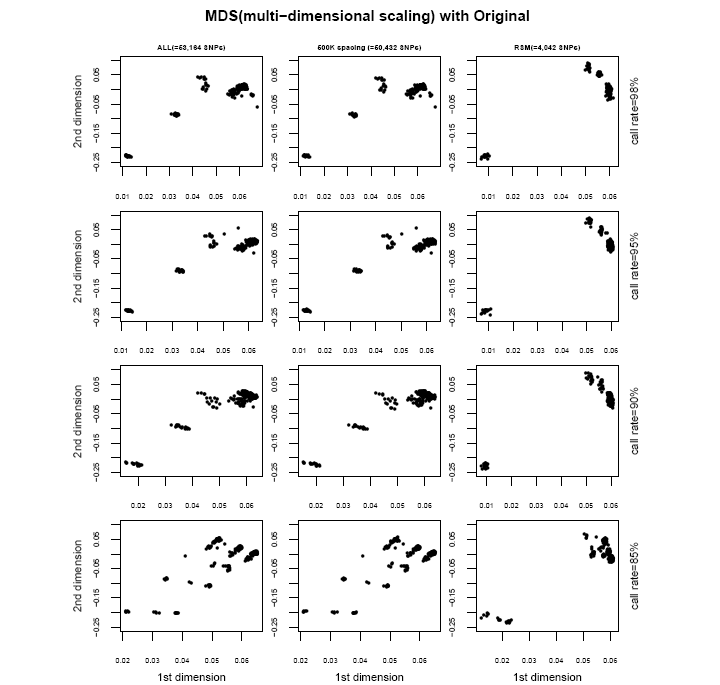

Supplement: Figure S2 — MDS with recombination hotspot (RH) filtering. (0.06 MB TIF) [file pone.0011855.s004.tif]

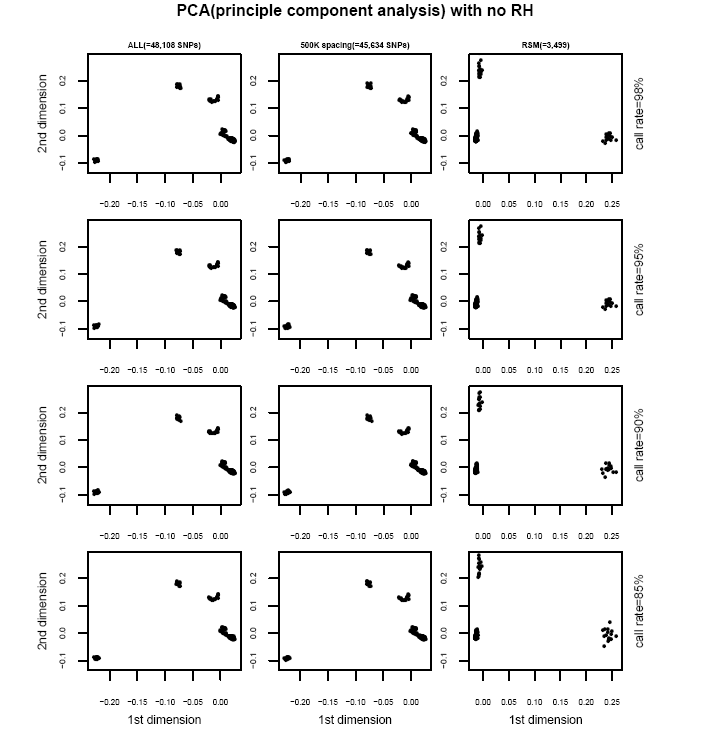

Supplement: Figure S3 — PCA without recombination hotspot (RH) filtering. MDS plots with SNPs after QC, QC+500KB spacing between SNPs, and only regional specific markers (RSM) are shown in columns. Different genotype call rates (98% to 85%) are shown in rows. (0.06 MB TIF) [file pone.0011855.s005.tif]

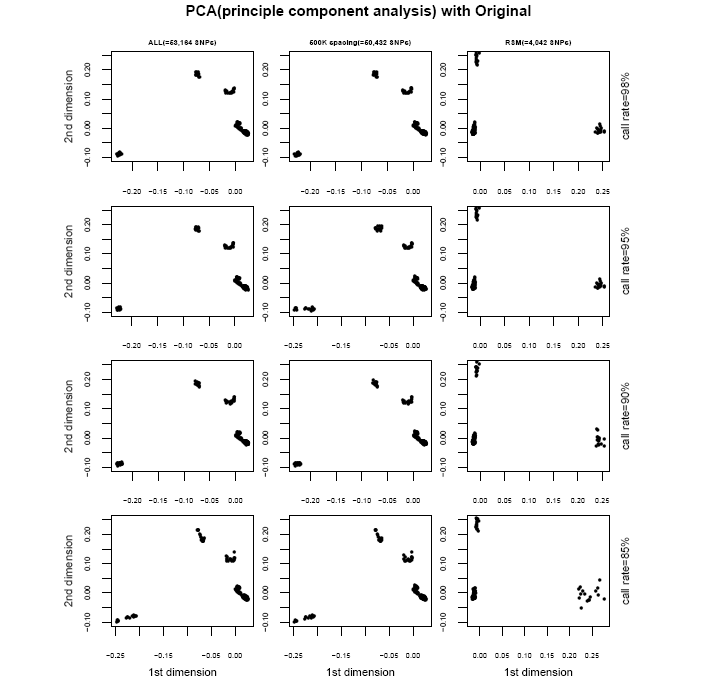

Supplement: Figure S4 — MDS with recombination hotspot (RH) filtering. (0.06 MB TIF) [file pone.0011855.s006.tif]
